# Beyond the Gut Feeling: A Retrospective Analysis of Adult Foreign Body Ingestion, Associated Risk Factors, and Treatment Outcomes

**DOI:** 10.7759/cureus.96270

**Published:** 2025-11-06

**Authors:** Imad Elkhalil, Hussein W Khudhur, Hisham Hurreiz, Mustafa Jameel

**Affiliations:** 1 Department of Education, Sheikh Khalifa Medical City, Abu Dhabi, ARE; 2 Department of General Surgery, Burjeel Hospital, Abu Dhabi, ARE; 3 Department of Radiology, Sheikh Khalifa Medical City, Abu Dhabi, ARE; 4 Department of General Surgery, Sheikh Khalifa Medical City, Abu Dhabi, ARE

**Keywords:** endoscopic approach, esophageal impaction, psychiatric co-morbidities, suicidal and accidental ingestion, swallowed foreign body

## Abstract

Background

Adult foreign body ingestion (FBI) shows different patterns from pediatric cases and poses variable needs for endoscopic or surgical intervention, depending on object type, risk factors, and anatomical location. International guidelines recommend urgent removal for high-risk objects, such as sharp items, magnets, or batteries, and early endoscopy for selected esophageal impactions. To our knowledge, this study, for the first time in the United Arab Emirates (UAE), investigated the epidemiology, risk factors, management strategies, and outcomes of adult FBI cases at a tertiary hospital, comparing accidental and intentional ingestions.

Methods

A single-center, retrospective cohort of adults (≥18 years) presenting with confirmed or suspected gastrointestinal FBI between January 2017 and December 2023 was reviewed. Data on demographics, psychiatric and substance-use history, object type, location, management, complications, and outcomes were collected. The primary outcome was the need for endoscopic or surgical intervention. Comparative analyses used Fisher’s exact and Mann-Whitney U tests.

Results

Forty-six patients with gastrointestinal FBI were included (25 accidental and 21 intentional). The median age was 31 years (interquartile range, or IQR: 27-43), and 52% were male. Intentional ingestion was strongly associated with psychiatric illness (95.2%) and substance-use disorder (85.7%). Overall, 33 of 46 (71.7%) patients required an intervention (endoscopy or surgery): 23 of 25 (92.0%) accidental vs. 10 of 21 (47.6%) intentional (Fisher p = 0.001). High-risk objects (sharp items and batteries) predominated among intentional ingestions, which had longer hospital stays (median: 5 vs. 1 day; p < 0.001).

Conclusion

Accidental FBI typically involves esophageal food impaction requiring prompt endoscopy, while intentional ingestion occurs mostly in psychiatric patients, involves high-risk objects, and exhibits recurrent, resource-intensive patterns. These findings highlight the need for structured triage pathways, early psychiatric involvement, and preventive strategies within mental health frameworks to reduce recurrence and hospitalization burden.

## Introduction

Foreign body ingestion (FBI) is a common challenge encountered in emergency and gastroenterological practice [1]. The most commonly affected are children aged six months to six years [2]. In adults, foreign bodies typically involve food-related items, with fish bones and chicken bones being the most commonly encountered [2]. Additionally, adults with psychiatric illnesses, neurological disorders, or gastrointestinal abnormalities represent a significant at-risk group. Up to 20% of cases necessitate urgent endoscopic evaluation, and fewer than 1% require surgical intervention [3]. Although current evidence shows a generally benign course, FBI can be associated with significant morbidity and, in rare cases, mortality, thus warranting timely recognition and evidence-based management [2].

The spectrum of ingested foreign bodies differs markedly by circumstance, with intentional objects including sharp razors, needles, and batteries, and accidental cases mostly involving bones, food boluses, and dental prostheses [2,4]. The most frequent complication of these ingestions is esophageal impaction, which warrants urgent endoscopic evaluation to prevent mucosal injury and airway compromise [5]. Batteries can cause rapid tissue perforation, local necrosis, and burns [6], while sharp objects pose a higher risk of perforation, potentially leading to life-threatening complications such as mediastinitis, peritonitis, or abscess formation [7].

Multiple current guidelines address the approach to managing FBIs, most notably the guidelines published by the European Society of Gastrointestinal Endoscopy (ESGE) and the American Society for Gastrointestinal Endoscopy (ASGE) [7,8]. Both recommend that high-risk situations warrant immediate endoscopic evaluation, preferably within two to six hours of presentation [7,8]. These high-risk situations include complete esophageal obstruction, batteries, or sharp-pointed objects [7,8]. The management of other, less dangerous foreign bodies - whether urgent or non-urgent - can be determined by the type, size, location, and symptoms of the patient [7,8]. The protocols set by these guidelines are generally conservative, including observation rather than immediate removal for asymptomatic patients with blunt objects that have passed through the esophagus [7,8]. However, there remains considerable variability when it comes to non-endoscopic management strategies, particularly for intentional ingestions and for patients with psychiatric risks [9].

To address current evidence gaps in the UAE, this retrospective study aimed to assess the epidemiology, risk factors, management approaches, and outcomes of FBI in adults by systematically comparing clinical presentations, intervention requirements, and outcomes between patients with accidental and intentional ingestions, with a particular emphasis on comparing outcomes between these two groups. The study was conducted at a tertiary-level hospital and spanned six years. This approach helps optimize guideline-based management and identify potential opportunities for targeted psychiatric and preventive measures.

Study aims and objectives

The primary objective of this study was to assess the epidemiology, risk factors, management approaches, and outcomes of FBI in adults presenting to a tertiary care center over six years.

The specific objectives included (1) to characterize the demographic and clinical profiles of patients with FBI; (2) to compare object types, their common presentations, and the differences in anatomical locations between accidental and intentional ingestions; (3) to determine the rates of endoscopic and surgical interventions, stratified by ingestion intent; (4) to evaluate clinical outcomes, including complications, hospital length of stay, and recurrence patterns.

## Materials and methods

Study design and setting

This is a single-center, retrospective cohort study of adult patients aged ≥18 who presented to Sheikh Khalifa Medical City (SKMC), a tertiary-level center in Abu Dhabi, United Arab Emirates, between January 1, 2017, and December 31, 2023. This facility serves as a major regional referral center for gastroenterological emergencies, providing comprehensive endoscopic and surgical management. Data were extracted retrospectively from electronic medical records and radiological imaging databases. Ethical approval was obtained from the Institutional Review Board (Approval no. REC-07.10.2025 (RS-943)), and informed consent was waived due to the project's retrospective and minimal-risk nature.

Case identification

Patients were selected via an electronic search of emergency department records using predefined keywords: “foreign body,” “coin,” “magnet,” “battery,” and “food bolus.” This initial search was cross-checked with endoscopic procedure logs and International Classification of Diseases (ICD) diagnostic codes. Inclusion criteria included age ≥18 years and presentation with clinician-documented or imaging-confirmed gastrointestinal foreign body impaction/ingestion during the study period. Exclusion criteria included pediatric patients and those with essential clinical records that were missing.

Data collection

Data were extracted into a structured data collection form developed specifically for this study (Appendix 1). The dataset included demographic variables such as age, sex, and age category (adults defined as ≥18 years and elderly as ≥65 years). Clinical history variables comprised the presence of a documented psychiatric illness, substance-use disorder, or prior episodes of FBI. Presentation characteristics included the patient’s symptoms on arrival, the time elapsed between ingestion and hospital presentation (in hours), and the imaging modality utilized, whether plain radiography or computed tomography. Foreign-body characteristics were documented under standardized categories - bone, food bolus, battery, magnet, sharp object, dental prosthesis, or other - and the anatomical location reported on imaging was recorded as esophagus, stomach, small bowel, or other. Management variables encompassed the approach taken (conservative observation, endoscopic intervention, or surgical intervention), the time from presentation to endoscopic procedure (in hours), and whether endoscopic removal was successful. Outcome variables included in-hospital complications such as perforation, bleeding, mediastinitis, or sepsis; hospital length of stay (in days); intensive care unit admission; and 30-day readmission related to the foreign body. Ingestion intent was classified based on medical record documentation of deliberate self-harm behavior and psychiatric history (intentional) versus unintended ingestion during eating with food-related objects (accidental). High-risk objects were defined as batteries, multiple magnets, and sharp-pointed items (e.g., needles, razor blades, pins, or large nails) [7,8]. An “intervention” was defined as any endoscopic or surgical procedure performed during the index admission with the intent to remove the object, while “complications” referred to any adverse event arising from the foreign body or its removal.

Outcome measures

The primary outcome included the requirement for intervention - whether endoscopic or surgical - during the patient’s index admission, with the intent to remove the foreign body.

The secondary outcomes included intervention success (complete foreign body removal), inpatient complications (perforation, bleeding, mediastinitis, or sepsis), hospital length of stay, intensive care unit admission, and 30-day readmission for foreign body-related complications.

Statistical analysis

Data were analyzed for the entire cohort and stratified by ingestion intent (accidental vs. intentional). Continuous variables were summarized as medians with interquartile ranges (IQRs) due to non-normal distributions. Categorical variables were reported as counts and percentages, with 95% Wilson confidence intervals for key proportions. Group comparisons used Fisher's exact tests for categorical variables and the Mann-Whitney U test for continuous variables. All statistical tests were two-sided, with p < 0.05 considered statistically significant.

Statistical analyses were conducted using Python (version 3.10.12, Python Software Foundation, Wilmington, DE, USA) and R (version 4.2.3, R Foundation for Statistical Computing, Vienna, Austria).

## Results

Cohort characteristics

A total of 46 patients presenting with FBI met the inclusion criteria during the six-year study period. The cohort was stratified based on intention: 21 patients (45.7%) were classified as intentional, and 25 patients (54.3%) were classified as accidental.

The overall median age was 31 years (IQR: 27-43 years), and 24 patients (52.2%) were male. Significant clinical and demographic differences existed between the intentional and accidental groups (Table 1).

**Table 1:** Baseline demographic and clinical characteristics by ingestion intent Comparison of demographic features, psychiatric comorbidities, and risk factors between patients with accidental (n = 25) and intentional (n = 21) foreign body ingestion. Data are presented as median (interquartile range, or IQR) for continuous variables and n (%) for categorical variables. p-values were calculated using Fisher’s exact test for categorical variables and the Mann-Whitney U test for continuous variables.

Variable	Accidental (n = 25)	Intentional (n = 21)	p-value
Median age (IQR), years	40 (31-50)	28 (25-30)	0.006
Male, n (%)	9 (36.0)	15 (71.4)	0.021
Psychiatric history, n (%)	3 (12.0)	20 (95.2)	<0.001
Substance use disorder, n (%)	2 (8.0)	18 (85.7)	<0.001
Prior foreign body ingestion, n (%)	2 (8.0)	14 (66.7)	<0.001
High-risk object, n (%)	2 (8.0)	20 (95.2)	<0.001

Patients with intentional FBI were notably younger than those with accidental ingestion (median age 28 vs. 40 years, p = 0.006) and were more frequently male (71.4% vs. 36.0%, p = 0.021). Although documented psychiatric illnesses had a prevalence in half of the total cohort, they were drastically more prevalent among the intentional group (20/21, 95.2%) compared to the accidental group (3/25, 12.0%; p < 0.001). Furthermore, substance use disorder prevalence showed a similar pattern, appearing in nearly half the overall cohort (20/46, 43.5%) but was more prevalent among the intentional group (18/21, 85.7%) than in the accidental group (2/25, 8.0%; p < 0.001). A history of prior FBI was reported in over one-third of the overall cohort (16/46, 34.8%) and was more prevalent in the intentional group (14/21, 66.7%) compared to the accidental group (2/25, 8.0%; p < 0.001).

Object types and anatomical location

Object types differed strikingly by ingestion intent (Table 2): (1) accidental cases were mostly bones (n = 14) and food boluses (n = 7), with most lodged in the esophagus (21/25, 84.0%); (2) intentional cases were predominantly sharp objects (n = 13) and batteries (n = 7), and these objects were predominantly located beyond the esophagus (21/21, 100.0% in stomach or distal sites) compared to accidental ingestions.

**Table 2:** Distribution of foreign body types by ingestion intent Distribution of ingested object types categorized by ingestion intent (n = 46). Values represent the number of cases in each category. Sharp objects included glass shards, razors, and blades. “Other” included nail clippers.

Foreign Body Type	Accidental (n = 25)	Intentional (n = 21)	Total (n = 46)
Battery	0	7	7
Bone	14	0	14
Dental prosthesis	2	0	2
Food bolus	7	0	7
Pin/Needle	2	0	2
Sharp object	0	13	13
Other	0	1	1

Management and outcomes

Overall, 33 of 46 patients (71.7%) required an intervention (endoscopic or surgical removal) during the index admission (Table 3). Intervention rates were significantly different between groups, with 23 of 25 accidental cases (92.0%) versus 10 of 21 intentional cases (47.6%; p < 0.001). 

**Table 3:** Management approach and clinical outcomes by ingestion intent Management modalities and procedural outcomes for 46 patients with foreign body ingestion. Endoscopy success represents the successful removal of those undergoing endoscopy. Values are n (%). p-values were calculated using Fisher’s exact test for categorical variables, and the Mann-Whitney U test for continuous variables.

Variable	Accidental (n = 25)	Intentional (n = 21)	p-value
Conservative management, n (%)	2 (8.0)	11 (52.4)	<0.001
Endoscopy, n (%)	22 (88.0)	10 (47.6)	<0.001
Surgery, n (%)	1 (4.0)	0 (0.0)	0.341
Endoscopy success, n (%)	15 (68.1)	5 (50.0)	0.001
Total interventions, n (%)	23 (92.0)	10 (47.6)	<0.001

Among the 33 patients who required intervention, only a single patient required surgical intervention, while the remaining 32 patients (97.0%) underwent endoscopic removal. Endoscopic removal was successful in the majority of cases, predominantly in the accidental group (15/22, 68.2%) compared to the intentional group (5/10, 50.0%; p < 0.001). 

In-hospital complications were rare, occurring in 2 of 46 patients (4.3%). These included one esophageal perforation, which required surgical repair, and a case of post-procedure infection, which was managed with prolonged antibiotic therapy. 

**Figure 1:**
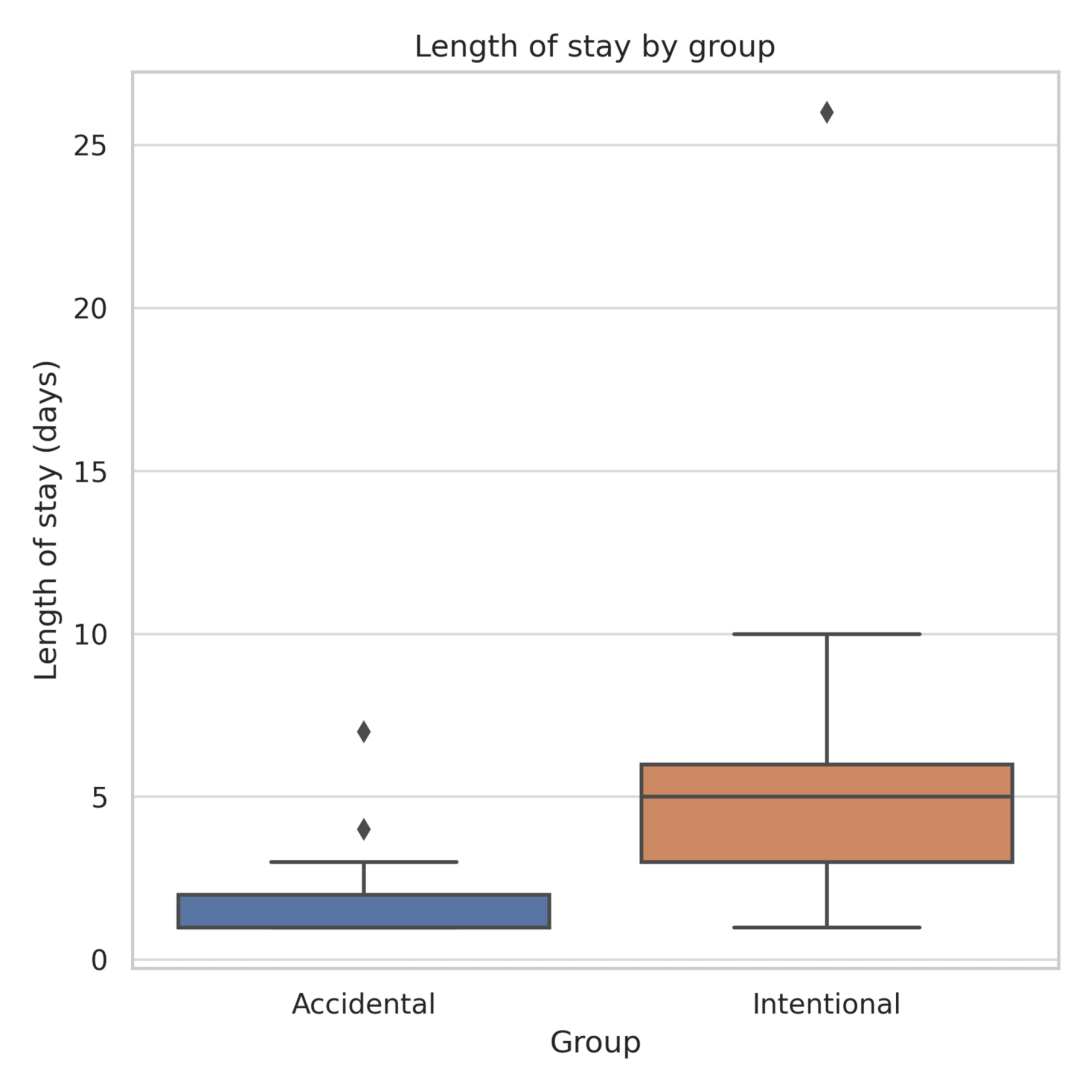
Hospital length of stay by ingestion intent Comparison of hospital length of stay (in days) between patients with accidental and intentional foreign body ingestion. The figure displays median values with interquartile ranges (IQRs) for each group. Patients with intentional ingestion had a significantly longer hospital stay (median 5 days, IQR 3-6) compared to those with accidental ingestion (median 1 day, IQR 1-2; p < 0.001). Statistical comparison was performed using the Mann-Whitney U test.

The hospital length of stay differed significantly between the two groups (Figure 1). Patients with intentional ingestion had a median length of stay of five days (IQR: 3-6 days; p < 0.001), while those with accidental ingestion had a median length of stay of one day (IQR: 0-1 day; p < 0.001).

## Discussion

This retrospective study examined a cohort of 46 adult patients with a history of FBI at a tertiary care center over six years, identifying two demographically and clinically distinct phenotypes. Accidental ingestions, mainly involving food-related objects that lodge in the esophagus, occurred primarily in older adults and required urgent endoscopic intervention in over 90% of cases. In contrast, intentional ingestions predominantly affected younger patients with documented psychiatric illnesses and substance use disorders, involved the ingestion of sharp objects and batteries, and were associated with higher recurrence risk, longer hospital stays, and lower intervention rates. These findings stress the importance of targeted psychiatric preventive strategies and differentiated management protocols.

The demographic and clinical profiles of accidental FBI in our cohort align closely with previous reports [2,10]. The predominance of food-related objects that result in accidental ingestion and their concentration in the esophagus has been described in large international series [2,10,11]. Our intervention rate for accidental ingestions (92%) is higher than the 20% reported in some studies [2,12], reflecting the higher incidence of esophageal impaction in our cohort. This difference may also relate to the prevalence of dietary patterns, local demographics in the UAE, and the case mix of the referral center. The favorable outcomes, with low complication rates (4.3%), align with guideline-based endoscopic management as well as current guidelines and recommendations set by the ESGE and ASGE for early intervention in esophageal foreign bodies [7,8].

The intentional ingestion in our cohort demonstrated a strikingly different profile, with nearly universal psychiatric comorbidities (95.2%) and a high rate of substance use disorder (85.7%). These findings mirror international studies describing a strong association between deliberate FBI and mental health disorders, specifically schizophrenia, substance abuse disorders, and borderline personality disorder [12]. The repetitive nature of this behavior has been reported in psychiatric literature and aligns with the finding that 66.7% of patients had prior FBI [13]. Notably, our intentional group had a significantly lower intervention rate (47.6% vs 92.0%), despite the higher-risk objects. This is partially explained by the fact that most objects were distal to the esophagus and by the effectiveness of the conservative approach in such cases, as previously reported in a retrospective cohort study [14]. The five-fold extended hospital stay of intentional ingestion patients, compared to accidental ingestion patients (median 5 vs. 1 day), emphasizes the need for psychiatric evaluation, social work planning, and disposition planning. This burden is rarely quantifiable in prior studies [15].

These highlighted findings have several important clinical implications. First, the clinically and demographically distinct profiles support risk-stratified triage protocols. Intentional ingestions can be prevented with early psychiatric evaluation, and immediate intervention is not always medically indicated [14]. In contrast, accidental ingestions can benefit from urgent endoscopic removal and airway protection. Second, managing psychiatric patients purely with medical approaches explains the higher recurrence rates. This highlights the need for an integrated care model that involves social work, psychiatry, and harm-reduction strategies, such as close follow-ups on an outpatient basis and crisis intervention plans [12-14]. Third, our data suggest that if the high-risk object is distal to the esophagus at the time of presentation, conservative management with serial imaging may be sufficient [14]; however, this requires validation in a larger cohort. Finally, healthcare systems should comprehend the burden associated with recurrent intentional FBIs and implement preventive psychiatric strategies.

This study has several strengths, including standardized data extraction, systematic identification of data across multiple sources, and detailed characterization of both intentional and unintentional ingestion of foreign bodies over six years. However, several limitations need to be acknowledged. The single-center design and the sparsity of data hinder multivariable modeling due to collinearity. Second, the retrospective nature of the study allows for misclassification bias, especially regarding the intent of ingestion when documentation is incomplete. Third, the study lacks follow-up data beyond 30 days, which hinders proper assessment of recurrence rates and the effectiveness of psychiatric interventions. Finally, as a tertiary care hospital in the UAE, our cohort may differ from that of community hospitals in terms of demographics, case complexity, and access to subspecialty care. Moreover, to our knowledge, this represents the first comprehensive analysis of adult FBI in the UAE, addressing a significant gap in regional literature and providing locally relevant data to inform clinical practice and resource allocation.

Future studies, including multicenter studies with larger sample sizes, can help develop a validated risk prediction model for complications and intervention requirements. Randomized controlled trials evaluating structured psychiatric interventions are needed to reduce the recurrence rate in high-risk patients. Finally, qualitative research investigating patient perspectives and challenges in obtaining psychiatric follow-up, versus standard care, may help identify preventive strategies for this vulnerable population. 

## Conclusions

This single-center, retrospective study identified two distinct clinical profiles of FBI among adults over a six-year period. Accidental FBIs were predominantly related to food objects and esophageal impactions, ultimately requiring endoscopic removal in the majority of cases. In comparison, intentional ingestion of foreign bodies was primarily prevalent in patients with psychiatric illnesses and substance use disorder. It involved high-risk objects, ultimately resulting in longer hospital stays and higher recurrence rates.

## Appendices

Appendix 1

**Table 4:** Data collection

record_id	mrn_hashed	age_years	sex	nationality_region	date_of_presentation	time_from_ingestion_to_presentation_hours	intent	setting	risk_esophageal_disease	risk_dentures_or_poor_dentition	risk_neurological_disorder	risk_psychiatric_history	risk_substance_use_disorder	risk_prior_esophageal_surgery_or_radiation	risk_prior_FBI_history	risk_pediatric_developmental_delay	vitals_abnormal	symptoms_drooling	symptoms_dysphagia_odynophagia	symptoms_retrosternal_pain	symptoms_cough_or_wheeze	symptoms_vomiting	symptoms_respiratory_distress	symptoms_abdominal pain	site_suspected	fb_type_category	fb_size_length_mm	fb_size_width_mm	radiopaque	imaging_done	complication_at_presentation	time_to_endoscopy_hours	endoscopy_performed	endoscopy_urgency	endoscopic_modality	endoscopic_tools_used	endoscopic_success	surgery_required	surgery_type	nonoperative_management	passed_spontaneously	length_of_stay_days	icu_admission	complications_in_hospital	post_endoscopy_stricture_dilation	mortality_in_hospital	re_presentation_within_30_days	disposition	follow_up_attended_within_8_weeks	follow_up_findings	missing_data_notes	abstractor_initials
